# The effect of different variables on push-out tests in 3D-printed oval and round-shaped root canals: a methodological study

**DOI:** 10.2340/aos.v84.42958

**Published:** 2025-02-13

**Authors:** Tuba Gok, Guzide Cankaya, Bilge Hakan Sen

**Affiliations:** a Department of Endodontics, School of Dentistry, Firat University, Elazig, Turkey; b Fethi Sekin Oral and Dental Health Center, Elazig, Turkey; c Emeritus Professor, Izmir, Turkey

**Keywords:** 3D printing, artificial root canal, dislocation resistance, push-out test, root canal treatment

## Abstract

**Objective:**

This study aimed to evaluate the effect of slice thickness (ST), plunger size (PS), shape and region of the root canal on push-out tests using standardized artificial root canals.

**Materials and methods:**

Two teeth with round and oval root canal anatomy were selected using cone beam computed tomography. Teeth were prepared, scanned with micro computed tomography and stereolithography data were obtained. Seventy-two round and 72 long oval artificial root canals were produced using a 3D printer. Root canals were obturated, then divided into two main groups (oval-round) and further divided into six subgroups (*n* = 12) according to ST (1, 1.5, and 2-mm) and PS (0.5, 0.75, and 1-mm). Push-out tests were performed and dislocation resistance values were calculated. The data were analyzed using the ANOVA two-way test (*p* = 0.05).

**Results:**

Different STs showed similar results in oval canals (*p* > 0.05). 1-mm ST showed higher results in round canals (*p* < 0.05). There was a significant difference between 0.75 and 1-mm PSs (*p* < 0.05). Middle and coronal regions showed similar results in oval canals (*p* > 0.05), and coronal region showed lower results in round canals (*p* < 0.05).

**Conclusion:**

ST, PS, root canal shape and region variables affected the dislocation resistance of core material in standardized root-filled canals.

## Introduction

The push-out tests have been widely used in endodontic research to evaluate the bond strength of different materials. Although the push-out tests cannot be correlated with actual endodontic results to measure the adhesion of filling materials to the root canal wall, they are important for the reliable ranking of the quality of filling materials and techniques [1]. However, differences in dentine structure and variations in methodologies lead to concerns in interpreting results and preclude comparing the results of push-out bond strength studies [1, 2].

Methodological variables such as sealer, core material, root filling technique, tooth type, tooth portion, slice thickness (ST) [3], plunger size (PS), load velocity and storage time affect the resistance to bond strength [3–5], which cause a wide range of values in terms of both bond strength and size of standard deviations [1, 2]. In literature, several studies have investigated the variables affecting the findings in push-out tests [4, 6, 7], but differences in the dentine structure used in these studies were also evaluated as a confounding factor [1, 8].

In laboratory studies, one of the main limitations of push-out tests is the difficulty of establishing a reliable basis because of the complex anatomy and variable morphology of dentine [9]. Since the microstructure and mechanical behavior of dentin vary [10], it is almost impossible to achieve complete standardization in real teeth. [11]. In recent years, some methodological studies have begun to use 3D technologies by producing samples with standard root canal anatomy to overcome this situation [12–14]. 3D printing technology has become widely accepted and rapidly developed in dentistry [15] and, studies have shown that the area and volume of natural root canals are not significantly different from 3D-printed canals [16, 17].

The use of 3D-printed artificial root canals allows the researchers to design balanced experimental groups and similar conditions within the root canals by eliminating factors such as root canal anatomic variations, morphological diversity of dentine and different irrigation and preparation procedures [1]. Therefore, the importance of utilizing standard test methods has been emphasized for comparability and reproducibility of different material/application combinations [18].

To the best of our knowledge, no study has used artificial root canals to standardize the methodology of push-out tests. For this purpose, standardized artificial root canals were employed in this study to evaluate the influence of ST, PS, the shape of root canal (oval vs. round) and root canal region variables on push-out tests. The null hypothesis was that the different ST, PS, shape of the cross-sectional root canal (oval vs round) and root canal region variables do not affect the dislocation resistance of the filling material.

## Materials and methods

### Sample size calculation

According to a previous study [4], the minimum sample size required to detect a significant difference should be at least 12 in each group, considering type I error (alfa) of 0.05, power (1-beta) of 0.85, and effect size of 0.58 (WSSPAS; Web-Based Sample Size & Power Analysis Software [19]).

### Sample selection, preparation and 3D printing

Ethical approval was obtained from the Firat University Non-Interventional Ethics Committee (process no. 2021/07 – 24), following the principles and guidelines of the Declaration of Helsinki for medical research involving human subjects. A maxillary canine with a round root canal and a distal root of a mandibular molar with a long oval root canal, the ratio of long to short canal diameter to be ≥2, but ≤4 [20], were selected by imaging with cone-beam computed tomography (Planmeca; ProMax 3D Mid, Helsinki, Finland) to confirm the root canal anatomy. The teeth did not have more than one apical foramen, caries, or micro-cracks on the root surfaces, internal/external resorption, root canal treatment, or immature root apex. The coronal segments of the teeth were removed using Struers Accutom-3 machine (Struers; Copenhagen, Denmark) with a diamond disc (Struers) under continuous water irrigation.

After the working length determination, the roots were instrumented up to ProTaper Universal F5 file (Dentsply Maillefer; Ballagiues, Switzerland). After each file, the root canals were irrigated with 2 mL 2.5% sodium hypochlorite (Werax; Izmir, Turkey). 2 mL 17% ethylenediaminetetraacetic acid (Werax), 5 mL 2.5% sodium hypochlorite and 2 mL distilled water was used as final irrigation. The root canals were dried using size 50 paper points (Diadent; Chongju, Korea).

**Figure 1:**
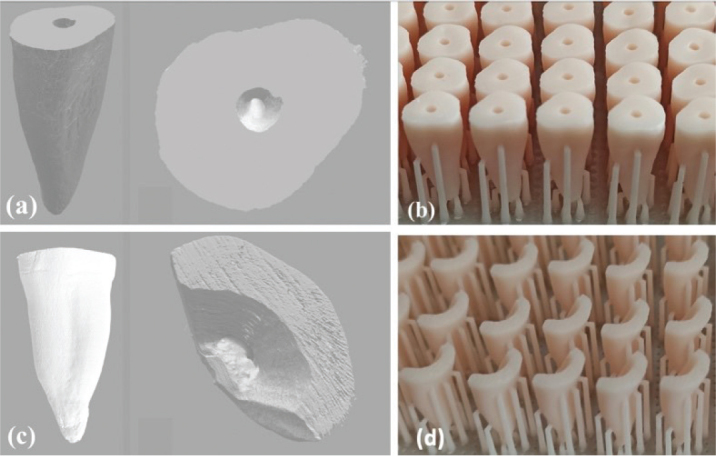
Stereolithography (STL) format images of roots scanned with Micro-CT: the root with round (a) and oval-shaped canals (c), 3D-printed root images of round (b) and long oval-shaped (d) root canals.

The roots were then scanned with a SkyScan micro-computed tomographic system (SkyScan 1272; Bruker micro-CT, Kontich, Belgium) at a 10-μm ST. The X-ray tube was operated at 80 kV and 125 mA. The scanning procedure was performed at 0.4-degree rotation angles with an aluminum filter. The CTAn software (Skyscan; Aartselaar, Belgium) was used to obtain 3D high-resolution images of roots and converted to stereolithography (STL) files after images were reconstructed (NRecon v.1.6.9; Bruker micro-CT) (Figures 1a and 1c).

The STL files were transferred to a Digital Light Processing (DLP) 3D printer (DentaFab Sega 3D printer; 3bFab, İstanbul, Turkey). Seventy-two round and 72 long oval artificial root replicas were manufactured in 50-μm resolution using a ceramic-based 3D-printing dental model resin (Powerresins Model Resin; 3bFab) (Figures 1b and 1d). The 3D printing process was applied with 0.05 mm Z and 0.047 mm XY precision. The round canal model consisted of 368 layers and the oval canal model was 352. The 3D-printed roots were then cleaned with alcohol in an ultrasonic bath for 2 minutes. The post-curing process was performed under ultraviolet light for 4 minutes.

### Obturation and slicing of artificial roots

All artificial root canals were filled using the single-cone technique. The tip of the EndoActivator (Dentsply Tulsa Dental Specialties; Tulsa, OK) was adjusted to be 2 mm less than the artificial root canal length. The AH Plus sealer (Dentsply De Trey; Gmbh, Konstanz, Germany) was inserted into the root canals by in and out motion of EndoActivator with an amplitude of 3-mm for 5 seconds. The F5 ProTaper Universal (Dentsply Maillefer) gutta-percha cone was coated with AH Plus sealer and smeared into the root canal at working length. The excess gutta-percha was removed and the vertical compaction was performed with a compatible plugger at the root canal orifice. Temporary filling material was placed at the canal orifices. (Coltosol; Coltene, Altst€atten, Switzerland). The roots were checked with periapical radiography for the possible presence of voids. A single operator has completed the experimental procedures of root canal filling. The artificial roots were then divided into two main groups according to the root canal shape (round and long oval). The groups were further divided into six subgroups (*n* = 12) according to the ST (1, 1.5 and 2-mm) and PS (PS) (0.5, 0.75 and 1-mm) for each root canal shape group and stored at 37°C – 100% humidity.

**Figure 2:**
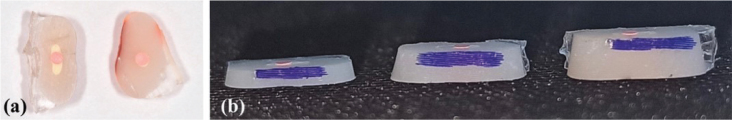
Representative images of (a) section samples obtained from artificial root canals with oval and round-shaped canals, and (b) slice thicknesses as 1, 1.5 and 2-mm.

After 1 week, the artificial roots were fixed on acrylic resin rods. The cutting process was performed horizontally with a low-speed diamond saw (Struers) at 350 rpm under water cooling, considering the disc thickness (0.3 mm). For the ST groups, 1, 1.5 and 2-mm slices were obtained from the middle part of the round and oval roots (Figures 2a and 2b). Two-mm slices were obtained from the coronal part of the round and oval roots for the PS groups. The coronal surface of each slice was marked with an indelible marker. The apical and coronal surfaces of each slice were checked with an operating microscope (OMS 2360; Zumax, Suzhou, China). Fourteen sections with voids were excluded and replaced with new sections. The thickness of each slice was measured with a digital caliper (±0.1 mm) (Max-Extra Professional Tools; Guangzhou, China). The images of each slice were acquired with a stereomicroscope (Olympus BX43; Olympus Co, Tokyo, Japan) under 10X magnification. The major and minor perimeters (the root canal area on both surfaces of the section) of oval and round canals were measured with AxioVision Rel. 4.8 software (Zeiss, G€ottingen, Germany) before push-out tests. Since the slices with gaps were excluded, the areas of the root canals of each slice were equal to the root canal filling areas.

### Push-out test and assessment

**Figure 3:**
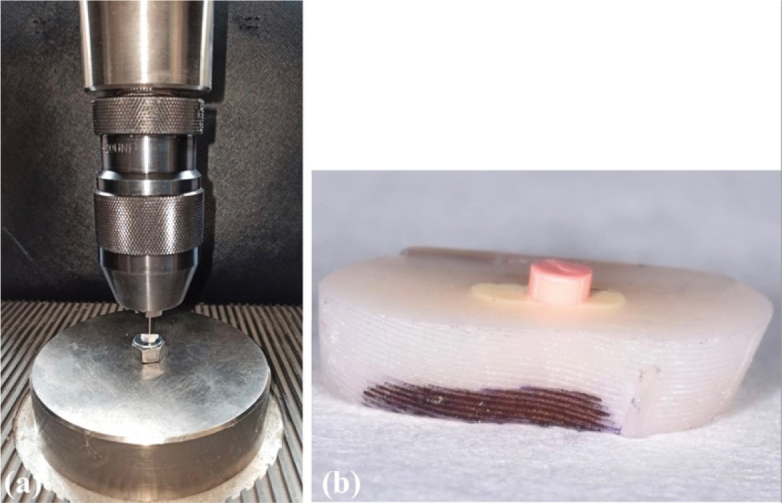
Representative images of (a) the push-out test design and (b) gutta-percha dislodged after the push-out test.

The load was applied by a cylindrical stainless steel plunger tip of 0.75-mm diameter for the ST groups and 0.5, 0.75 and 1-mm diameters for PS groups over the gutta-percha in an apical–coronal direction (Figure 3a). The plunger tip was positioned to cover as much as possible of the root filling and avoid any contact with the canal walls. The push-out test was performed on Shimadzu Autograph AG-X universal testing machine (Shimadzu; Tokyo, Japan) at a crosshead speed of 0.5 mm min^−1^ until gutta-percha dislocation occurred (Figure 3b). A load (N) × dislodgement curve was plotted automatically during the test. Another single operator has performed the experimental procedures of push-out tests.

The resulting force (N) was converted to a megapascal (MPa) unit by dividing the lateral area of the intracanal material and dislocation resistance was calculated. The lateral areas of round and oval canals were calculated according to Pereira et al.’s study [21].

### Data presentation and statistical analysis

The dislocation resistance data were normally distributed (Shapiro-Wilk, *p* > 0.05) and had homogeneity of variance (Levene’s test, *p* > 0.05). Therefore, parametric tests were used. The ANOVA two-way test was used to evaluate the effect of ‘root canal shape and ST’ and ‘root canal shape and PS’ on the dislocation resistance values. The Post-hoc LSD test was used for both ST and PS groups for multiple comparisons. The independent sample *t*-test was used to evaluate the influence of the root canal region on the dislocation resistance values. Statistical tests were performed using SPSS for Windows, Version 26.0 (IBM Corp., Armonk, NY), and the cut-off level for significance was set at α = 5%.

## Results

Tables 1, 2 and 3 show the mean, standard deviation and *p* values of oval and round canals tested at different STs, PSs and root canal regions.

**Table 1:** Mean and standard deviation (SD) of the dislocation resistance (MPa) of the tested oval and round canals in different slice thicknesses (*n* = 12) (The plunger size is constant in all groups / 0.75 mm).

	1-mm	1.5-mm	2-mm			

Group/Slice Thickness	Mean ± (SD)			*p*-value*	*p*-value**	
Oval					(1 mm – 1.5 mm)	0.206
	0.27 (0.08)^Aa^	0.21 (0.05)^Aa^	0.27 (0.10)^Aa^	*p >* 0.05	(1 mm – 2 mm)	0.929
					(1.5 mm – 2 mm)	0.177
Round					(1 mm – 1.5 mm)	**< 0.001**
	0.52 (0.20)^Ab^	0.31 (0.09)^Bb^	0.27 (0.09)^Ba^	***p <* 0.05**	(1 mm – 2 mm)	**< 0.001**
					(1.5 mm – 2 mm)	0.309
*p*-value***	**< 0.001**	**0.026**	0.914			

Different uppercase letters indicate significant differences between the slice thickness in each line, whilst lowercase letters indicate the significant differences between the canal shapes in each column (*p* < 0.05).

* The *p*-value of the multiple comparisons of oval or round canals at different slice thicknesses.

** The *p*-value of the pairwise comparisons of oval or round canals at different slice thicknesses.

*** The *p*-value of the pairwise comparisons of oval and round canals at the same slice thicknesses.

**Table 2:** Mean and standard deviation (SD) of the dislocation resistance (MPa) of the tested oval and round canals in different plunger sizes (*n* = 12) (The slice thickness is constant in all groups / 2 mm).

	0.5-mm	0.75-mm	1-mm			

Group/Plunger Size	Mean ± (SD)			*p*-value*	*p*-value**	
Oval					(0.5 mm – 0.75 mm)	0.353
	0.30 (0.07)^ABa^	0.27 (0.10)^Aa^	0.41 (0.10)^Ba^	***p <* 0.05**	(0.5 mm – 1 mm)	0.06
					(0.75 mm – 1 mm)	**<0.01**
Round					(0.5 mm – 0.75 mm)	0.175
	0.24 (0.05)^ABa^	0.19 (0.04)^Ab^	0.33 (0.12)^Bb^	***p <* 0.05**	(0.5 mm – 1 mm)	0.22
					(0.75 mm – 1 mm)	<**0.01**
*p*-value***	0.092	**0.036**	**0.030**			

Different uppercase letters indicate significant differences between the plunger sizes in each line, whilst lowercase letters indicate the significant differences between the canal shapes in each column (*p* < 0.05).

* The *p*-value of the multiple comparisons of oval or round canals at different plunger sizes.

** The *p*-value of the pairwise comparisons of oval or round canals at different plunger sizes.

*** The *p*-value of the pairwise comparisons of oval and round canals at the same plunger sizes.

**Table 3:** Mean and standard deviation (SD) of the dislocation resistance (MPa) of the tested canals at different root canal regions (*n* = 12). (The plunger size and slice thickness are constant in all groups / respectively 0.75 mm and 2 mm).

	Middle	Coronal	

Group/Region	Mean ± (SD)	*p*-value	
Oval	0.27 (0.10)^a^	0.27 (0.10)^a^	0.938
Round	0.27 (0.09)^a^	0.19 (0.04)^b^	**0.021**

Different letters indicate significant differences between the root canal region in each line (*p* < 0.05).

In ST groups, there was no significant difference in dislocation resistance of different STs in oval canals (*p* > 0.05). Samples with 1-mm ST showed statistically significantly higher dislocation resistance compared to the samples with 1.5 and 2-mm STs in round canals (*p* < 0.05). There were statistically significant differences between oval and round canals at 1 and 1.5-mm STs (*p* < 0.05); 1 and 1.5-mm STs showed higher dislocation resistance in round canals. There was no statistically significant difference between oval and round canals at 2-mm ST.

In PS groups, the dislocation resistance was higher at 1-mm PS both in both oval and round canals, but the difference was only statistically significant between 0.75 and 1-mm PSs (*p* < 0.05). There was no statistical difference in dislocation resistance in oval and round canals at 0.5-mm PS (*p* > 0.05). The oval canals showed no statistical difference in the middle and coronal sections (*p* > 0.05), while the dislocation resistance was statistically higher in the middle sections in round canals.

## Discussion

This study showed that ST in round canals, PS in both oval and round canals, and root canal region in round canals had an impact on the dislocation resistance in artificial root canals; therefore, the null hypothesis was partially rejected.

Many studies have evaluated the push-out bond strength of materials to the root canal dentine [6, 22, 23]. However, the variations, particularly in methods, prevent comparing the findings of these studies. Several studies have examined the effects of test variables on push-out bond strength [4, 22, 24, 25]. However, the lack of pooled data availability in this respect limits reliable and further assessment of the role of each variable [3]. Although natural teeth are still the standard practice in ex-vivo studies due to their properties such as natural tissue hardness, morphology, color, texture and radiodensity [26], they have disadvantages such as collection difficulties, ethical considerations, potential cross-infection risk and most importantly standardization [11]. In addition, the usage of natural teeth has potential confounders such as tooth age, storage time and medium, and the distribution of sclerotic dentine, which can lead to differences in outcomes between studies [1]. The use of well-standardized 3D-printed root canal spaces can overcome biological, chemical and physical variances of root dentine and provide similar conditions for all samples [1]. In addition, they are available immediately in a sufficient number [27], and in ex-vivo studies, the morphological standardization achieved has a major impact on the reliability of results [28]. Therefore, this study compared the effect of ST, plunger diameter, cross-sectional root canal shape and root canal region variables on dislocation resistance using artificial standardized root canals. The standardization of samples ensured homogeneity between groups and prevented the risk of selection bias.

In this study, the single cone technique was preferred instead of lateral compaction because it has been reported that fewer cones increase the dislocation resistance [3]. In addition, maximum standardization was achieved in all samples by using a single cone. STs and PSs were selected by the study results of Chen et al. [5] to calculate the dislocation resistance formula with suitable samples. In all groups, the PSs were smaller than 0.85 times the filler diameter but were not small as to puncture the filler material and the samples’ thickness was larger than 0.6 times the filler. Therefore, to use plungers in three different diameters in the PS groups, the slices were taken from the coronal parts where the canal diameter was larger.

In the present study, there was no significant difference in dislocation resistance of different STs in oval canals (*p* > 0.05). On the other hand, in round canals, samples with 1-mm ST showed significantly higher resistance compared to 1.5 and 2-mm STs (*p* < 0.05). These results conflict with a previous meta-regression analysis, which reported that STs > 1 mm increased the resistance to dislodgement compared to the resistance of slices with a thickness of 1 mm or less [3]. They explained this situation by the higher frictional resistance of materials retained in the root canal. As the ST increases, the maximum force (N) dislocating the filling material is likely to increase due to sliding friction. However, in the formula applied to calculate the dislocation resistance (MPa), the maximum force is divided by the lateral area of the section. In other words, in a sample with a larger ST, the greater maximum force will be divided into a greater area, and in a sample with a smaller ST, the less maximum force will be divided into a smaller area. As a result, when we formulate the force, it is expected that there should not be a significant difference at different slice thicknesses. Therefore, it is theoretically questionable to explain the greater dislocation resistance in slices thicker than 1 mm with greater sliding friction because the slice thicknesses of each of these analyzed studies were not compared within themselves. In addition, when comparing different studies, the different formulas used to calculate the bond strength also affect the resistance values. In line with all this, it is more likely that factors such as the tooth used, root canal volume, filling material diversity, storage time, PS and sliding friction at different interfacial roughness [29] are more effective on dislocation resistance.

Interesting findings in the present study demonstrated that there was no difference in dislocation resistance of different STs in oval canals as predicted (*p* > 0.05), but round canals showed inconsistent results by the dislocation resistance decreased while the ST increased. Unfortunately, as there is no other study on the relation between ST and dislocation resistance, it is not possible to compare this finding with a previous study. These inconsistent results in round canals may be related to the damage to the gutta-percha sealer adhesion that may occur during the sectioning procedure.

The present study found that the round canals showed higher dislocation resistance than oval canals at 1 and 1.5 mm STs (*p* < 0.05). This result was similar to that of previous studies, which evaluated the effect of root canal shape on bond strength [30, 31]. In these studies, the dislocation resistance was tested at 1-mm ST and it has been reported that the reduced thickness of the sealer/bonding material in round canals results in lower polymerization shrinkage and subsequently lower polymerization stress, so the bond strength was greater than in oval canals [30–32]. There was no significant difference in oval and round canals at 2-mm STs in our study. Pereira et al. [21] stated that oval or long oval sections have 3 to 4 times more sealant in the middle and coronal thirds than round canals, making the polar areas of the root filling more susceptible to disruption when the load is applied. The reason for the lack of difference in 2-mm ST may be that the susceptibility to disruption of the polar areas decreases as the section thickness increases. Based on these findings, assuming that thinner sealer thickness causes lower polymerization stress, it may be more appropriate to prefer root canal filling methods such as warm filling methods that will reduce the sealer thickness in oval canals, especially for resin-based materials.

Different PSs showed statistically significantly different values in oval and round root canals in the present study (*p* < 0.05). Our study’s findings were similar to Nagas et al.’s study [4], which investigated the effect of plunger diameter (0.75, 1 and 1.25-mm) on the push-out bond strength values of different root filling materials. Although there was no statistically significant difference in the 0.75 and 1-mm diameters (except in one group), which had the same diameters in both studies, the dislocation resistance values increased while the plunger diameter increased as in our study. There was no statistically significant difference in dislocation resistance between 0.5-mm plunger diameters in our study (*p* > 0.05), but as stated in the previous study, the plunger diameter should not be small to avoid notching the plunger into the material surface, which could affect the dislocation resistance [33].

In this study, no statistical difference was found in dislocation resistance in the middle and coronal sections of oval canals (*p* > 0.05). The middle sections showed higher dislocation resistance in round canals (*p* < 0.05). Previous studies reported conflicting results about the bond strength in different regions of root canals. In some studies, the bond strength values decreased from the coronal to the apical region [34, 35]. However, other studies found that the bond strength was highest for the apical and lowest for the coronal section [22, 36]. The differences in findings may have been caused by the use of natural teeth, which were not standardized in these studies. In a study similar to our study, the effect of root canal cross-sectional shape on single cone root filling bond strength was evaluated in natural teeth [21]. Although oval canals showed similar findings to our study, the contrary results were obtained in round canals. In natural teeth, the difference between dentinal tubules’ diameter and number and intertubular dentin areas in different root canal regions [37] can also cause these conflicting results.

Although clinical studies are of great importance, bond strength in the root canal can be evaluated with mechanical tests in in-vitro or ex-vivo studies. And by providing comparison of different materials, it can guide the choice of materials that perform well in clinical material selection. This study has shown that some variables in methodologies can affect the results, therefore, if in-vitro or ex-vivo studies are to be used as a reference for the selection of any material from a clinical perspective, care should be taken to ensure that the methodology in the study is well standardized.

This study had the following limitations. Firstly, we evaluated the dislocation resistance by using artificial root canals. An artificial root canal does not reflect actual clinical conditions due to its microstructural features, such as its micro-hardness being different from dentine or the absence of dentinal tubules that may influence adhesion and dislocation resistance in natural teeth. However, as mentioned earlier, the main purpose of this laboratory study was not to evaluate the actual endodontic findings but to evaluate the effects of methodological variables on dislocation resistance. Secondly, the dislocation resistance was performed via gutta-percha dislocation from the sealer. It has been reported that the nature of the root canal filling (sealer alone or sealer + core material) may also play an important role in the measurement of dislocation resistance [8]. Therefore, further laboratory studies are required to determine the dislocation resistance of just sealer from the root canal wall by using standardized root canal anatomy. Thirdly, due to the nature of the push-out test design, the sectioning of samples may also negatively affect the gutta percha-sealer or sealer-dentine adhesion during the procedure. Therefore, methods such as finite element analysis may also be preferred. The strengths of our study included that the groups were well-standardized; hence the variables could be evaluated reliably.

## Conclusion

In summary, the shape of the root canal, ST, PS and root canal region variables partially affected the dislocation resistance of filling material. The present study demonstrated that the methodological variables are of great importance in reliably comparing studies with the push-out bond strength and standardization of variables can lead to a more comparable and reproducible analysis of the dislocation resistance of root filling materials.

## Data Availability

The data supporting this study’s findings are available from the corresponding author upon reasonable request.
